# A clinical perspective of sepsis-associated delirium

**DOI:** 10.1186/s40560-016-0145-4

**Published:** 2016-03-23

**Authors:** Ryosuke Tsuruta, Yasutaka Oda

**Affiliations:** Acute and General Medicine, Yamaguchi Graduate School of Medicine, 1-1-1 Minamikogushi, Ube, Yamaguchi 755-8505 Japan

**Keywords:** Delirium, Coma, Acute brain dysfunction, Confusion Assessment Method for the Intensive Care Unit (CAM-ICU), Intensive Care Delirium Screening Checklist (ICDSC), Sepsis-associated encephalopathy (SAE)

## Abstract

The term sepsis-associated encephalopathy (SAE) has been applied to animal models, postmortem studies in patients, and severe cases of sepsis. SAE is considered to include all types of brain dysfunction, including delirium, coma, seizure, and focal neurological signs. Clinical data for sepsis-associated delirium (SAD) have been accumulating since the establishment of definitions of coma or delirium and the introduction of validated screening tools. Some preliminary studies have examined the etiology of SAD. Neuroinflammation, abnormal cerebral perfusion, and neurotransmitter imbalances are the main mechanisms underlying the development of SAD. However, there are still no specific diagnostic blood, electrophysiological, or imaging tests or treatments specific for SAD. The duration of delirium in intensive care patients is associated with long-term functional disability and cognitive impairment, although this syndrome usually reverses after the successful treatment of sepsis. Once the respiratory and hemodynamic states are stabilized, patients with severe sepsis or septic shock should receive rehabilitation as soon as possible because early initiation of rehabilitation can reduce the duration of delirium. We expect to see further pathophysiological data and the development of novel treatments for SAD now that reliable and consistent definitions of SAD have been established.

## Introduction

The Pneumonia Severity Index, which was developed in the USA, includes five key clinical factors: pulse rate, respiratory rate, systolic blood pressure, body temperature, and mental status [1]. Altered mental status was also cited in the diagnostic criteria for sepsis proposed by Levy et al. [2]. Mental confusion, rather than altered mental status, was used to assess the severity of community-acquired pneumonia in another study [3]. Although these studies did not clearly define altered mental status or mental confusion, these states may be indicative of delirium because an acute change in mental status is a major feature of delirium [4].

**Fig. 1:**
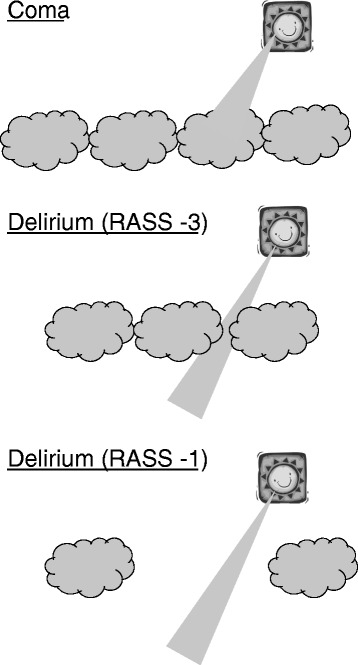
Illustration of acute brain dysfunction (coma and delirium). The *sun* represents the investigator. The *sunbeam* represents the interview or tests (i.e., CAM-ICU) used to assess inattention. The *clouds* (i.e., RASS score of ≤−4) represent cover on some levels of arousal required to maintain cognition. *RASS −3* the patient opens their eyes or moves in response to a voice but does not make eye contact. *RASS −1* the patient is not fully alert but opens their eyes and makes eye contact, which is sustained for more than 10 s in response to a voice

**Fig. 2:**
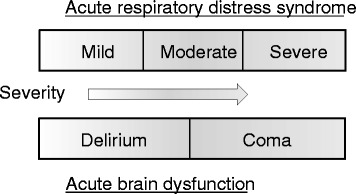
The relationships between coma and delirium in contrast to acute respiratory distress syndrome

The American Psychiatric Association’s Fifth Edition of the Diagnostic and Statistical Manual of Mental Disorders (DSM-5) revised the diagnostic criteria for delirium [5]. All states of altered arousal, except coma, are included in the definition of delirium (Fig. 1). Delirium and coma occurring in patients with critical illness are referred to as acute brain dysfunction (Fig. 2) [6]. The terms septic encephalopathy or sepsis-associated encephalopathy (SAE) are often used in Japan. However, “delirium” is used globally instead of“encephalopathy” in current diagnostic manuals such as DSM-5 and the International Classification of Diseases (ICD-10). In the present review, we have used the terms sepsis-associated delirium (SAD) or infectious delirium. SAD is considered a diffuse cerebral dysfunction caused by the systemic inflammatory response to an infection without evidence of a central nervous system infestation (i.e., infection) [7].

## Review

### Pathophysiology of SAD

#### Neuroinflammation

**Fig. 3:**
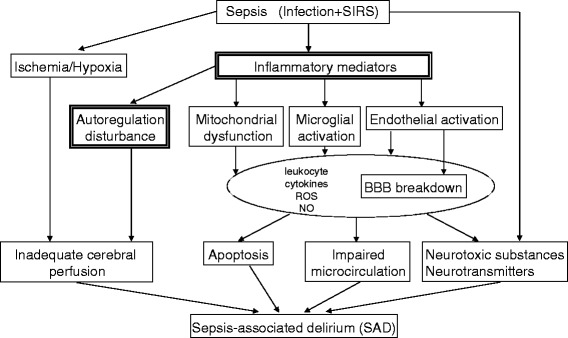
The mechanism of sepsis-associated delirium. *Double-lined boxes* correspond to the clinical evidence reported in the main text. *SIRS* systemic inflammatory response syndrome, *ROS* reactive oxygen species, *NO* nitric oxide

The systemic inflammatory response is a key phenomenon in critical illnesses and may progress to organ dysfunction, including the brain. Systemic inflammation and endothelial activation frequently occur during critical illnesses and may augment cytokine transport across the blood–brain barrier (BBB) [8], BBB disruption [9], and the infiltration of leukocytes and cytokines into the central nervous system [10]. These events may result in ischemia and neuronal apoptosis, which may present clinically as delirium [11]. These mechanisms have been investigated using animal models [7, 12, 13] (Fig. 3).

Some recent clinical studies have sought to standardize the detection of delirium in critically ill patients. In a prospective cohort study [14], interleukin 6 (IL-6) concentrations were higher in delirious patients than those in comatose patients [14]. Another study revealed that, after adjusting for covariates, lower plasma concentrations of matrix metalloproteinase-9 (MMP-9) and protein C and higher concentrations of soluble tumor necrosis factor receptor-1 (sTNFR1) were associated with increased risk of delirium [15]. These results suggest that inflammation and coagulation may play important roles in the development of delirium in patients with critical illness. We also assessed delirium and obtained blood samples at two times: extubation (0 h) and 24 h after extubation (24 h) [16]. We found that the serum concentrations of procalcitonin and IL-6 were significantly higher in the delirious patients than those in the non-delirious patients at 24 h, but not at 0 h. Crucially, these studies showed an association between the onset of delirium and circulating biomarkers in critically ill patients in both infectious and non-infectious conditions. However, there are no clinical studies describing an association between SAD (i.e., infectious delirium) and biomarkers, even though much data have been accumulated in animal models of sepsis [17, 18].

Two important studies have examined the association between delirium and biomarkers in two different situations. The first study of intensive care unit (ICU) patients with or without infection/systemic inflammatory response syndrome (SIRS) revealed that IL-8 concentrations were significantly associated with delirium in infected patients, whereas the anti-inflammatory cytokine IL-10 and amyloid β_1-42/40_ were associated with delirium in non-infected patients [19]. The second study examined the association between delirium and biomarkers in critically ill patients with or without sepsis [20] and revealed that plasma sTNFR1, sTNFR2, adiponectin, and IL-1β concentrations were higher in delirious patients than those in non-delirious patients. However, sepsis did not modify the associations between these biomarkers and the presence of delirium.

#### Cerebral perfusion abnormalities

Several studies have measured cerebral perfusion in patients with SAD. Pfister et al. [21] reported that SAD was significantly associated with C-reactive protein (CRP), S-100β, and cortisol concentrations, but not with IL-6 concentrations, and that elevated CRP was significantly associated with disturbed autoregulation. The impairments in cerebrovascular autoregulation were subsequently investigated daily for 4 days after onset in patients with severe sepsis or septic shock [22]. Autoregulation was impaired in 60 % of patients on day 1, 59 % on day 2, 41 % on day 3, and 46 % on day 4. SAD was present in 76 % of patients. Impaired autoregulation on day 1 was also associated with the presence of SAD on day 4. These results suggest that dysfunction of cerebral autoregulation is a trigger for the development of SAD. Impaired autoregulation might result in cerebral hypoperfusion or hyperperfusion, as well as SAD.

In another study, reduced vascular reactivity (as measured by peripheral artery tonometry) was associated with a longer duration of delirium in a study of critically ill patients, in which 30 % of patients had severe sepsis on admission [23].

#### Neurotransmitter imbalances

Imbalances in several neurotransmitters, including the dopamine, γ-aminobutyric acid (GABA), and acetylcholine [24, 25] systems are also implicated in the etiology of delirium. Dopamine increases the neuronal excitability, while GABA and acetylcholine decrease neuronal excitability. An imbalance in one or more of these neurotransmitters results in neuronal instability and unpredicatable neurotransmission. Dopamine excess and acetylcholine depletion have been associated with delirium. Several studies in non-ICU settings have found a strong association between plasma anticholinergic activity (PAA), a measure of cholinergic blockade [26], and the development of delirium [27, 28]. Inflammation and cholinergic blockade, as measured by serum CRP and PAA, respectively, were associated with delirium in critically ill patients, and serum CRP and PAA were correlated with each other [29]. Another study in postoperative patients showed that the preoperative plasma cholinesterase activity was correlated with the changes in CRP and IL-6 concentrations after surgery in delirious patients [30]. Delirium was associated with impaired interactions between the cholinergic and immune systems. However, prophylactic, short-term administration of oral rivastigmine, a cholinesterase inhibitor, did not reduce the incidence of delirium in elderly patients for up to 6 days after elective cardiac surgery [31]. Therefore, owing to the multifactorial pathophysiology of delirium, interventions that only target the cholinergic system might be insufficient to prevent delirium.

### Diagnosis of SAD

#### Delirium screening tools

The 2013 American College of Critical Care Medicine (ACCM)/Society of Critical Care Medicine (SCCM) clinical practice guidelines for pain, agitation, and delirium (PAD) recommend that critically ill patients should undergo routine monitoring for the onset of delirium in the ICU using a validated tool [32]. The Confusion Assessment Method for the Intensive Care Unit (CAM-ICU) [33] and the Intensive Care Delirium Screening Checklist (ICDSC) [34] are the most valid and reliable tools for monitoring delirium in adult ICU patients [32].

The CAM-ICU, which was derived from DSM-4 criteria, evaluates four features of delirium: (1) acute changes or fluctuations in mental status from baseline, (2) inattention, (3) altered consciousness, and (4) disorganized thinking. The CAM-ICU is positive if the patient exhibits features 1 and 2 and either of features 3 or 4. Patients are defined as comatose if they respond only to physical/painful stimulation with movement but do not open their eyes (Richmond Agitation–Sedation Scale (RASS) [35] score, −4) or if they do not respond to verbal or physical stimuli (RASS score, −5) (Fig. 1). Delirium is defined as a response to verbal stimulation with eye opening (RASS score of −3 to +4) and a positive CAM-ICU value [4].

A change in the RASS score from −3 (the patient opens their eyes or move in response to a voice but does not make eye contact) to −4, a transition from delirium to coma, may be part of the continuum of progressively smaller levels of arousal. Alternatively, does this transition indicate a fundamental change in the pathophysiology of the acute brain dysfunction? [36]. The widespread administration of sedatives during mechanical ventilation of septic patients further complicates our understanding of acute brain dysfunction in such patients [36]. Subsequently, Patel et al. classified delirium into two categories: sedation-related delirium (delirium that abates shortly after sedative interruption) and persistent delirium [37]. After interrupting the patients’ sedatives, the investigator assessed whether the patients could follow four commands (squeeze the investigator’s hand, open their eyes, track an object with their eyes, or stick out their tongue) [38]. Patients who could not follow these commands after 2 h were classified as having persistent delirium. It was reported that the 1-year mortality rate was greater in patients with persistent delirium than that in patients without delirium or with sedation-related delirium [37]. In some studies, delirium was defined as a RASS score of >−3 [39] or >−2 [22].

The ICDSC, which is also derived from DSM-4 criteria, evaluates eight features of delirium: (1) altered consciousness; (2) inattention; (3) disorientation; (4) hallucinations, delusions, or psychoses; (5) psychomotor agitation or retardation; (6) inappropriate speech or mood; (7) disturbed sleep/wake cycles; and (8) symptom fluctuations [34]. The ICDSC, which incorporates the assessment of each feature over the course of the nursing shift, should not be used in heavily sedated patients (i.e., RASS score of ≤−4). The ICDSC is assessed based on the observations over the preceding 24 h. A total score of ≥4 denotes the presence of delirium, while total scores of 1–3 denote subsyndromal delirium [40]. The ICU mortality rates were 2.4, 10.6, and 15.9 % in patients without delirium, patients with subsyndromal delirium, and patients with delirium, respectively, defined according to ICDSC scores [40]. Therefore, the ICDSC allows ICU specialists to detect subsyndromal delirium, which is associated with intermediate adverse outcomes.

#### Electrophysiological testing

**Fig. 4:**
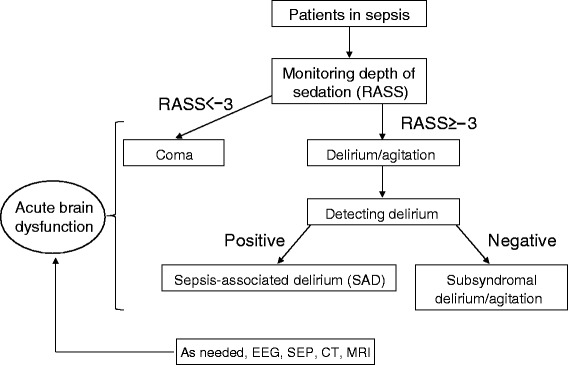
Diagnostic decision tree for acute brain dysfunction and sepsis-associated delirium (*SAD*). *RASS* Richmond Agitation–Sedation Scale, *EEG* electroencephalogram, *SEP* sensory evoked potential, *CT* computed tomography, *MRI* magnetic resonance imaging

Electrophysiological testing and neuroimaging are not always needed for the diagnosis of acute brain dysfunction or delirium in septic patients (Fig. 4). These tests should be considered in patients with persistent coma or delirium despite improvements in the functions of other organs and if the sedatives have been washed out.

The results of electroencephalograms (EEGs) are classified by severity based on the predominant waveform, including excessive *θ*, predominant *δ*, or triphasic waves, and suppression or burst suppression [41]. One study investigated the diagnosis of SAD by the CAM-ICU and the predominant EEG features on day 4 after a reduction of sedation to a RASS score of >−2 [22]. The authors reported that the diagnosis of SAD was not associated with EEG abnormalities. Furthermore, cerebrovascular autoregulation was not associated with EEG abnormalities.

Hosokawa et al. conducted a systematic review of EEG abnormalities in patients diagnosed with SAE or sepsis-associated brain dysfunction (SABD) [42]. The diagnostic methods were not reported in the systematic review and 5/17 studies were conducted before the introduction of the CAM-ICU and ICDSC in 2001. It is possible that SAE and SABD include coma and/or delirium in septic patients. The incidence of EEG abnormalities in septic patients ranged from 12 to 100 % for background abnormalities and from 6 to 12 % for triphasic waves. The authors concluded that EEG is a sensitive tool for the detection and diagnosis of SAE/SABD. A slowing of the normal *α* rhythm coinciding with the appearance of *θ* activity occurs in patients without evidence of encephalopathy and in patients with mild to moderate encephalopathy (e.g., confusion and delirium) and therefore reflects cortical dysfunction. More severe states of altered consciousness (e.g., stupor and coma) are associated with greater slowing together with increased *δ* activity, triphasic waves, and the more malignant burst suppression patterns that indicate impaired function of deeper brain structures, such as the basal ganglia and the diencephalon.

One study has sought to differentiate between coma and delirium in septic shock patients [43]. Coma was defined as a Glasgow Coma Scale (GCS) score of <8 in non-sedated patients or at 3 days after discontinuing sedation in previously sedated patients. Delirium was evaluated using the CAM-ICU. EEGs were analyzed using the Synek 5-point scale [44]. The median EEG grade was 3.5 (range 3.0–4.0) in coma and 3.0 (range 1.0–3.5) in delirium. An EEG grade of >3 was significantly more frequent in patients who died.

Compared with EEG, few studies have investigated sensory evoked potentials (SEP) in sepsis. The peak latency was significantly longer in patients with severe sepsis or septic shock than that in healthy controls, and it was correlated with the severity of sepsis [45]. However, the peak latency was not significantly different between patients with severe sepsis and those with septic shock. Although no studies have measured SEP in patients with SAD, a benefit of SEP is that it is not affected by sedation.

#### Neuroimaging

Brain computed tomography (CT) is often performed when septic patients display sudden changes in arousal or mental status, or persistent coma or delirium is unexplained by sedation. CT scans provide some information to aid the differential diagnosis, including focal brain infection, bleeding, and air embolism.

Magnetic resonance imaging (MRI) provides better and earlier information on brain damage, especially of the white matter and BBB. In a prospective study of 71 patients with septic shock, ischemic stroke and leukoencephalopathy were the two most frequent lesions [43]. Sharshar et al. hypothesized that leukoencephalopathy reflects BBB changes and identified it neuropathologically in a patient with diffuse leukoencephalopathy [46]. They also showed that an EEG grade of >3, as defined by Synek’s scale [44], was correlated with the presence of ischemic stroke or leukoencephalopathy on MRI in comatose or delirious patients. Therefore, they recommended that EEG should be done continuously or every day before MRI because of the risk of transport from the ICU to the MRI suite.

### Treatment of SAD

There are still no specific treatments for SAD. The treatment options for severe sepsis and septic shock are described in established guidelines [47], which recommend that continuous or intermittent sedation should be limited in mechanically ventilated septic patients and that the treatments should target specific titration endpoints. An a priori subgroup analysis of the Maximizing Efficacy of Targeted Sedation and Reducing Neurological Dysfunction (MENDS) study revealed that septic patients treated with dexmedetomidine had more days free of delirium than patients treated with a lorazepam-based sedation regimen [48]. However, lorazepam infusion is not available and dexmedetomidine at doses >0.7 μg/kg/h is not permitted in Japan.

Interestingly, two clinical trials have examined the association between stain use in an ICU and delirium in critically ill patients [49, 50]. Statins have pleiotropic effects on inflammation and coagulation. In the first study, statin exposure was independently associated with 2.28 times greater odds of being free of delirium [49]. In the second study, in which 55 % of patients had sepsis on day 1, statin exposure was associated with reduced risk of delirium among patients with sepsis on day 1 but not among patients without sepsis on day 1 [50]. A phase 2, randomized, placebo-controlled trial investigating whether simvastatin can reduce the risk of delirium in mechanically ventilated critically ill patients is ongoing (ISRCTN89079989).

Early physical and occupational therapy in mechanically ventilated patients, of whom 14 % had sepsis, decreased the number of days with delirium to half of that in the control group, even though the sedation regimen was identical in both groups (median 2 vs 4 days) [51]. Patients with severe sepsis or septic shock who display improvements and stabilization of their respiratory and hemodynamic states should receive rehabilitation as soon as possible. Delirium associated with sepsis or other causes was an independent predictor of the 6-month mortality rate [4], and the duration of delirium in the ICU was associated with long-term functional disability [52] and cognitive impairment [53, 54]. Furthermore, a cohort study revealed that severe sepsis was independently associated with persistent cognitive and functional limitations [55]. The risk of moderate to severe cognitive impairment tripled in septic patients. Taken together, these findings indicate that cognitive and physical rehabilitation is essential and should be initiated as soon as possible in the ICU and continued for as long as possible after discharge from the ICU.

## Conclusions

The pathogenesis of SAD (infectious delirium) and the features of this disorder in vivo are still unclear. Systemic inflammation and endothelial activation are common in critical illnesses and are associated with central nervous system disorders that may present clinically as delirium. Studies of patients with SAD have increased since the development of validated and reliable screening tools for delirium. However, when interpreting these studies, we should carefully assess their definitions of coma or delirium in septic patients. Finally, we believe that blood biomarkers, cerebrovascular autoregulation, electrophysiological, and neuroimaging tests are necessary in septic patients to establish consistent, pan-institutional definitions of coma/delirium.
